# Identifying bacterial and fungal communities associated with Fusarium-wilt symptomatic and non-symptomatic ‘Gros Michel’ banana plants in Ecuador

**DOI:** 10.3389/fcimb.2025.1572860

**Published:** 2025-06-23

**Authors:** Estefany M. Paredes Salgado, Fiama E. Guevara, Carlos Muentes, Francisco J. Flores, Freddy Magdama

**Affiliations:** ^1^ Departamento de Ciencias de la Vida y la Agricultura, Universidad de las Fuerzas Armadas (UFA) Universidad de las Fuerzas Armadas (ESPE), Sangolquí, Ecuador; ^2^ Escuela Superior Politécnica del Litoral (ESPOL), Centro de Investigaciones Biotecnológicas del Ecuador (CIBE), Guayaquil, Ecuador; ^3^ Centro de Estudios de Posgrado de la Universidad de las Fuerzas Armadas (ESPE), Sangolquí, Ecuador; ^4^ Agencia de Regulación y Control Fito y Zoosanitario (AGROCALIDAD), Quito, Ecuador; ^5^ Centro de Investigación de Alimentos, Facultad de Ciencias de la Ingeniería e Industrias, Universidad UTE, Quito, Ecuador; ^6^ Facultad de Ciencias de la Vida, Escuela Superior Politécnica del Litoral (ESPOL), Guayaquil, Ecuador

**Keywords:** microbiome analysis, *Musa acuminata* ‘Gros Michel’, *Fusarium oxysporum* f. sp. *cubense*, rhizome and rhizosphere soil, endophytic microbes, soil-borne fungal diseases

## Abstract

Fusarium wilt of banana (FWB), caused by *Fusarium oxysporum* f. sp. *cubense* (Foc), remains a critical threat to banana production worldwide. Despite the persistence of the disease in fields planted with susceptible cultivars such as ‘Gros Michel’, little is known about the microbial interactions influencing symptom development. In this study, we assessed the bacterial and fungal communities associated to symptomatic and non-symptomatic ‘Gros Michel’ bananas plants sampled in Ecuador banana fields affected by Foc race 1. We aimed to compare their diversity, composition, and to identify potential microbial taxa that could be active in disease suppression. Samples were collected from the pseudostem, rhizome, and rhizosphere, and analyzed through high-throughput sequencing of the 16S rRNA and ITS2 regions to characterize bacterial and fungal communities, respectively. Results revealed that non-symptomatic plants harbored significantly higher bacterial diversity, particularly in pseudostem and rhizome tissues, compared to symptomatic plants. Genera including, *Bacillus*, *Enterobacter*, *Paenibacillus*, *Pectobacterium*, *Herbaspirillum* and *Pseudomonas* were enriched in non-symptomatic tissues, suggesting a potential role in disease suppression. In contrast, symptomatic plants showed an increased abundance of genera such as *Klebsiella* and *Kosakonia.* Fungal community shifts were less pronounced, indicating that bacterial dynamics may play a more critical role in disease development. These findings shed light on the key microbial taxa associated with FWB-affected banana plants and the potential role of their microbiome to plant health and disease suppression.

## 1 Introduction

Bananas, are among the world´s leading food crops with a combined total production in 2022 of 135 million tons (Food and Agriculture Organization of the United Nations, 2023). More than 85% of all bananas produced are locally consumed, representing a key source of calories and nutrients for over 400 million people worldwide (Were et al., 2023). Bananas (including dessert bananas, plantains, and cooking bananas) are affected by numerous diseases and pests (Ploetz et al., 2015; Jones, 2019). However, Fusarium Wilt of Banana (FWB), caused by the soil-borne fungus *Fusarium oxysporum* f. sp. *cubense* (Foc), is the most destructive (Ploetz and Evans, 2015). Foc Race 1 severely affected the Gros Michel cultivar, which was the dominant banana variety for export during the mid-20th century (Ploetz et al., 2015). This led to significant economic losses and the eventual collapse of the Gros Michel-based banana industry (Roberts et al., 2024). In response to this crisis, the banana industry transitioned in the 1960s to the Cavendish cultivar, which was resistant to Foc Race 1. The widespread adoption of Cavendish bananas helped save the industry from collapse (Hou et al., 2022). Today, Cavendish cultivars represent 50% of global banana production and 95% of the international banana trade (García-Bastidas et al., 2022).

Foc tropical race 4 (Foc TR4), a set of isolates of race 4 capable of causing disease in tropical conditions, now threatens the Cavendish-based export industry and the production of other banana varieties as it keeps spreading despite all biosecurity measures implemented (Pegg et al., 2019). Currently, more than 27 countries have reported the incursion of Foc TR4 into their territory (Staver et al., 2020; Chittarath et al., 2022; Mejías et al., 2023). Frequently, these incursions are distinguished by the sudden onset of yellowing and wilting of foliage in plants as the primary external symptoms, eventually resulting in plant collapse (Ploetz et al., 2015; Viljoen et al., 2020). Once Foc TR4 invades the plant there is no cure, the fungus cannot be eliminated from the soil, fungicides are ineffective against it, and there is no resistant banana variety to replace the commercial Cavendish plantations (Ordonez et al., 2015; Garcia et al., 2018; Altendorf, 2019; Pegg et al., 2019).

For Ecuador, the world’s biggest banana exporter, Foc TR4 could pose a severe crisis since Cavendish banana exports account for a significant portion of the local economy with an estimated FOB (free of board) value of USD 3 billion, around 30% of its agricultural gross domestic product (Magdama et al., 2020). Moreover, with the presence of Foc TR4 in Colombia, Peru, and Venezuela, the entire LAC region is at high risk, as it supplies 28% of global banana production and over 80% of all banana exports (Martínez et al., 2023). Projected global losses attributed to Foc TR4 are estimated to reach 36 million tons of production by 2040 (Staver et al., 2020). However, the potential repercussions for consumers could be even more significant, with losses projected to soar to 235 billion if technology adoption to mitigate this pathogen is not pursued (de Figueiredo et al., 2023).

Breeding bananas conventionally is time-consuming, and genetic engineering may encounter various regulatory obstacles before adoption (Zorrilla-Fontanesi et al., 2020). However, in recent years, modern techniques like gene editing have garnered more attention as a viable alternative for producing contemporary bananas, including those resistant to pathogens like Foc TR4 (Tripathi et al., 2019). Meanwhile, alternative approaches to managing banana pathogens, particularly those focusing on soil health, are greatly needed. Hence, research into discovering potential biological control agents or manipulating microbial communities aimed at suppressing the pathogen in the soil has gained momentum (Berg et al., 2016).

The phytobiome plays a crucial role in plant development, health, and productivity as it intervenes in nutrient uptake, tolerance to stress, and induction of resistance to pathogens (Trivedi et al., 2020). Metagenomic analysis has allowed for significant improvements in our understanding of the phytobiome and soil microbiome and has provided tools to identify microorganisms that can be beneficial to the plant host (Cha et al., 2016). For instance, recent evidence shows common core bacteria in bananas worldwide, regardless of soil or genotype, which may play vital roles in plant health and growth (Birt et al., 2022). This increasing capacity for deep understanding of the holobiome- the host plant and its associated macro and microbiota, has also been proposed as a guide for improving breeding, and for the selection of agricultural management practices to enrich microbiota with desired functions (Mitter et al., 2019). Efforts in microbiome research are helping to develop novel solutions, such as utilizing new strains or combinations of strains, to enhance the effectiveness of microbial applications in diverse environmental settings (Callens et al., 2022). Although a wide range of environmental abiotic and biotic factors influence the performance and outcome of biological interactions, soon ecological models will enable us to predict the outcome of microbial inoculation better and to specifically select microbial strains or consortia with functional characteristics required in a specific environment (Schlaeppi and Bulgarelli, 2015; Mitter et al., 2019).

We hypothesize that distinct bacterial and fungal microbial profiles are significantly associated with either the promotion or suppression of FWB. These microorganisms likely colonize internal plant tissues and may influence disease outcomes through direct interactions with the pathogen or modulation of host responses (Kaushal et al., 2020a). The main objectives of this study were: (1) to compare the composition and diversity of bacterial and fungal communities associated with symptomatic and non-symptomatic ‘Gros Michel’ banana plants cultivated in Fusarium wilt-affected fields; and (2) to identify differentially enriched or depleted microbial taxa between symptomatic and non-symptomatic banana plants.

## 2 Materials and methods

### 2.1 Sites and samples collection

Samples were collected from two ‘Gros Michel’ banana farms located in Caluma Canton, Bolívar Province, Ecuador (Location 1: 1°37’26.0”S 79°18’39.6”W; Location 2: 1°37’29.4”S 79°18’29.1”W). The workflow of the current investigation is presented in **Supplementary Figure 1**. Samples of pseudostem, rhizome, and rhizosphere were collected from 10 plants per site, including 5 FWB- symptomatic plants, showing wilting, leaf yellowing, and internal discoloration, and 5 non-symptomatic. In total, 20 banana plants were sampled.

A cork borer, previously disinfected with 80% ethanol, was used to sample the pseudostem at 1.5 m above ground and the rhizome 0.2 m below ground. Plant tissue was sprayed with 1% citric acid upon sampling to prevent oxidation and transported in a 50 mL tube to the laboratory, where they were stored at 4°C until processing (Kaushal et al., 2020b). For rhizosphere analysis, approximately 100 grams of secondary roots containing soil were taken from each plant and handled under similar conditions.

### 2.2 DNA extraction and molecular identification of *Fusarium oxysporum* f. sp. *cubense*

DNA extraction from the pseudostem and rhizome samples was performed using the Wizard^®^ Genomic DNA Purification kit (Promega Corporation, U.S.A). Before processing, the pseudostem and rhizome were washed three times with distilled water to remove the 1% citric acid used for storage. For each sample, 500 mg of fresh tissue was ground in the presence of 50 mg of polyvinylpyrrolidone (Sahu et al., 2012), and the process continued following the Kit’s instructions. DNA extraction was performed in triplicate and pooled together to enhance sample representativeness. Rhizosphere samples were prepared by scraping 0.5 g of soil attached to the roots from which total DNA was extracted using the FastDNA™ Spin Soil kit (MP Biomedicals, U.S.A). Genomic DNA quality was assessed using a NanoDrop 2000 spectrophotometer (NanoDrop Technologies, U.S.A.) and by agarose gel electrophoresis. DNA samples were stored at −20°C until further use.

Pathogen presence in the collected samples was detected by amplifying the SIX9 and SIX1 genes, putative effectors secreted during the infection process of Foc race 1. The primer pairs used were FocSIX9 (F: 5’-GCAGTTGCGGCAATGGCT-3’; R: 5’-GCCCCATCTGGTATCCGACA-3’), and FocSIX1 (F: 5’-TGCATGACCACGAGTGTCC-3’; R: 5’-GCTTATGCTCAAGAGGCTGC-3’). PCR conditions were as follows: Initial denaturation at 94°C for 10 min; 30 cycles of 94°C for 45s, 58°C for 45s, and 72°C for 1 min; final extension at 72°C for 10 min (Magdama, 2017).

### 2.3 Amplicon sequencing

A total of 60 DNA samples (20 of each pseudostem, rhizome, and rhizosphere) were sent to BioSequence-EC (Quito, Ecuador) for high-throughput amplicon sequencing using the Illumina MiSeq platform. Libraries were generated through a two-step PCR process following the manufacturer’s protocol (Illumina, 2021). The V3-V4 region of the 16S rRNA gene was amplified using primers 341F (5’-CCTACGGGNGGCWGCAG-3’) and 806R (5’-GACTACHVGGGTATCTAATCC-3’) (Herlemann et al., 2011; Illumina, 2018) for the bacterial communities, while the internal transcribed spacer region 2 (ITS2) was amplified using primers ITS86F (5’-GTGAATCATCGAATCTTTGAA-3’) and ITS4 (5’-TCCTCCGCTTATTGATATGC-3’) (Vancov and Keen, 2009; Illumina, 2019) for fungal communities. Separate runs of sequencing were performed for each bacterial and fungal community. Approximately 70,000 paired-end reads (2 × 300 bp) were obtained per sample per run.

### 2.4 Amplicon sequence variant inference and taxonomic assignment

Amplicon sequencing reads were processed in the CEDIA High-Performance Computing cluster, based on the bioinformatic pipeline “Bioconductor Workflow for Microbiome Data Analysis” (Callahan et al., 2016, 2019) using R/RSudio (R Core Development Team, 2015). Quality control of the raw reads was performed in FastQC (Andrews, 2019), followed by Illumina adapter removal and trimming of low-quality reads using Trimmomatic v0.39 (Bolger et al., 2014).

Quality-filtered reads were processed following the DADA2 bioinformatic pipeline (Callahan et al., 2016). Inference amplicon sequence variants (ASVs) were performed with the argument pool=“pseudo” (Benitez et al., 2021). Sequences were filtered and trimmed based on quality profiles. 16S reads were truncated at 280 bp for forward reads and 220 bp for reverse reads, with filtering parameters detailed in the DADA2 Pipeline tutorial (version 1.16) (benjjneb.github.io/dada2/tutorial). ITS2 reads were not truncated, as recommended in the DADA2 ITS Pipeline Workflow (version 1.8) (benjjneb.github.io/dada2/ITS_workflow); however, a minLen=50 parameter was added to remove spurious low-length sequences. The SILVA (McLaren, 2020) and UNITE (Nilsson et al., 2019) databases were used for bacterial and fungal taxonomic assignments, respectively.

### 2.5 Bioinformatics and statistical analyses

A phylogenetic tree was created using the R package Phangorn (Schliep, 2011). Phyloseq objects containing an ASV table, sample data, a taxonomic assignment table, and a phylogenetic tree were created for the 16S and ITS2 data using phyloseq v1.22.3 (McMurdie and Holmes, 2013). Reads from unwanted taxa (i.e., host chloroplasts, and mitochondria) were removed from the 16S data set, and uninformative samples (i.e., samples with <1000 reads) were removed from both 16S and ITS datasets (Benitez et al., 2021). Data were normalized based on proportions (relative abundance) (Kuźniar et al., 2020). Three datasets were created for data analysis based on sample type (pseudostem, rhizome, and rhizosphere), and comparisons between plant symptomatology (S= symptomatic *vs*. N= non-symptomatic) were performed. Using the normalized data, relative abundance plots and heat maps were built by the apmvis2 package (Andersen et al., 2018), to identify symptomatology-related taxa. Heat trees were created through Metacoder (Foster et al., 2017), and differentially abundant taxa were identified using the DESeq2 R package (Anders and Huber, 2010).

Analyses of alpha and beta diversity were performed by the vegan and ampvis2 R packages (Dixon, 2003; Andersen et al., 2018). Alpha diversity metrics (observed richness, Shannon, Chao1, and Inverse Simpson index) were calculated for each sample using non-normalized data (Rausch et al., 2019). The effect of symptomatology (symptomatic or non-symptomatic) on alpha diversity metrics within each sample type (i.e., pseudostem, rhizome, and rhizosphere) was assessed using the non-parametric Kruskal-Wallis test, due to the non-normal distribution of the data. Beta diversity metrics (Bray-Curtis dissimilarity and weighted UniFrac) were calculated with normalized data and the distance matrices generated were used for principal coordinate analyses (PCoA) (Benitez et al., 2021). To test for differences in the bacterial and fungal communities between symptomatic and non-symptomatic samples, a PERMANOVA analysis with 1000 permutations was performed using the Bray Curtis distance matrix and the adonis2 function of the vegan R package (Luz, 2019).

## 3 Results

A total of 5,411,075 16S and 4,568,255 ITS2 amplicon sequencing reads were obtained from 60 samples. DADA2 processing of reads—including filtering, trimming, dereplication, merging, and chimera removal—resulted in 1,750,392 bacterial ASVs (32.35% of the input reads) and 3,824,572 fungal ASVs (83.37% of the input reads). After eliminating unwanted taxa, outliers, and less informative samples, 1,048,265 bacterial and 3,823,325 fungal ASVs were taxonomically assigned (**Supplementary Table 1**).

### 3.1 Taxonomic composition

**Figure 1:**
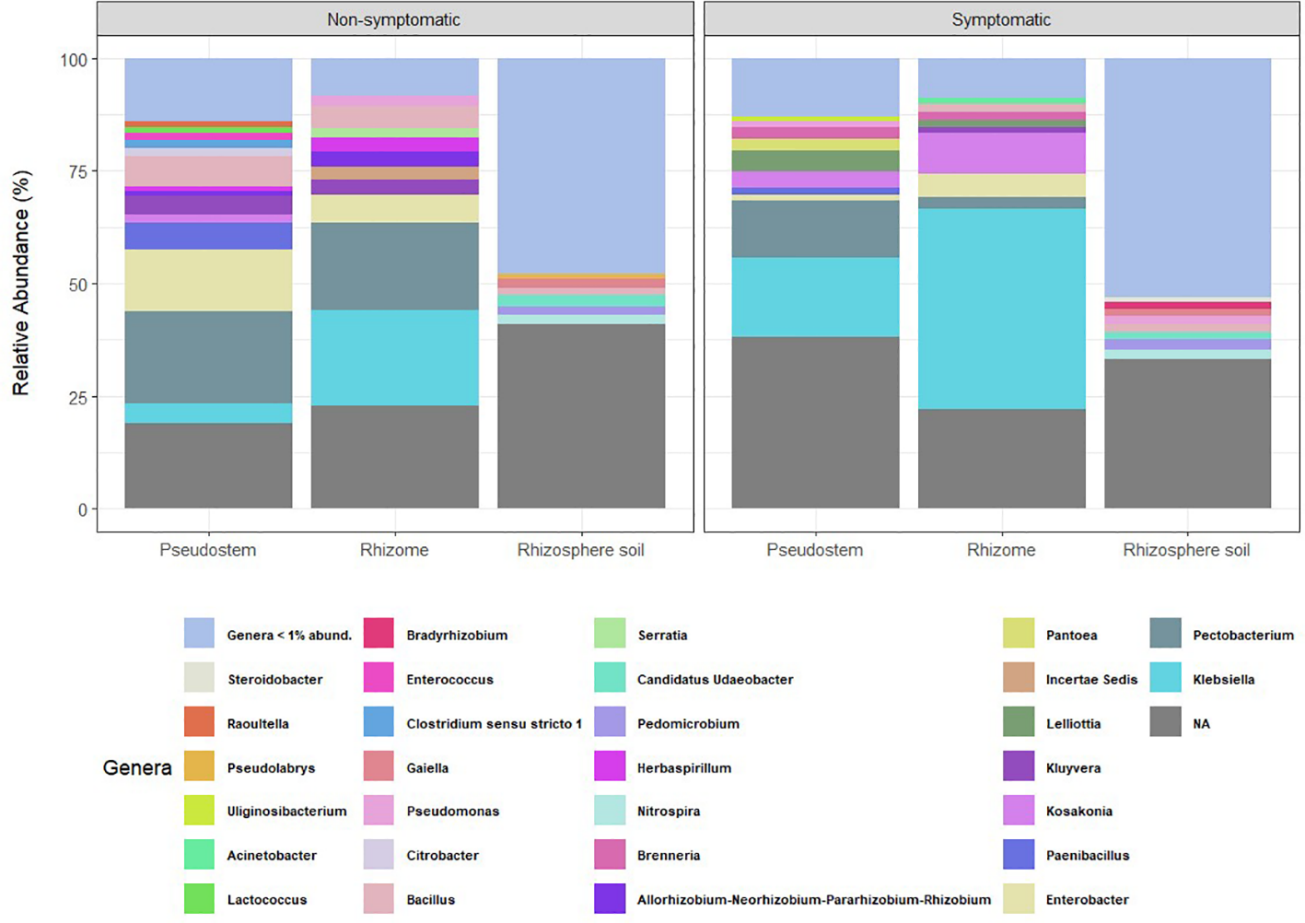
Relative abundance of bacterial genera categorized by environment. NA = ASVs with no taxonomic assignment at the genus level. Taxa with abundance < 1% are not shown.

Relative abundance analyses revealed that bacterial communities from the rhizome, pseudostem, and rhizosphere samples—regardless of symptomatology—were dominated by representatives of the *Proteobacteria*, followed by *Acidobacteriota* and *Firmicutes* phyla. However, the taxonomic profiles observed in rhizosphere samples, as shown in the relative abundance plots for phyla and genera, were markedly more diverse (**Figure 1**; **Supplementary Figure 2**). A higher proportion of ASVs without taxonomic assignment was also found in rhizosphere samples compared to those from rhizome and pseudostem.

The most abundant bacterial genera found in non-symptomatic pseudostem samples were *Pectobacterium* (20.4%), *Enterobacter* (13.6%), *Bacillus* (6.7%), and *Paenibacillus* (6%), while in non-symptomatic rhizome, the most abundant genera were *Klebsiella* (21.4%), *Pectobacterium* (19.4%), *Enterobacter* (6.1%), and *Bacillus* (4.8%). In the rhizosphere samples from non-symptomatic plants, Ca. *Udaeobacter* (2.5%), *Nitrospira* (2.1%), *Gaiella* (2%), and *Bacillus* (1.8%) were the most abundant. On the other hand, the top genera found in samples of symptomatic pseudostem were *Klebsiella* (17.7%), *Pectobacterium* (12,8%), *Lelliottia* (4.7%) and *Kosakonia* (3.6%), while in symptomatic rhizome, the most abundant genera were *Klebsiella* (44.5%), *Kosakonia* (9%), *Enterobacter* (5.3%) and *Pectobacterium* (2.5%). *Pedomicrobium* (2.3%), *Nitrospira* (2.2%), *Bacillus* (1.9%), and *Pseudomonas* (1.8%) exhibited the highest abundance in the rhizosphere samples collected from symptomatic plants. Heat map comparisons of relative abundances based on symptomatology revealed that pseudostem and rhizome tissues exhibited the most notable changes in bacterial profiles compared to rhizosphere samples (**Supplementary Figure 3**).

**Figure 2:**
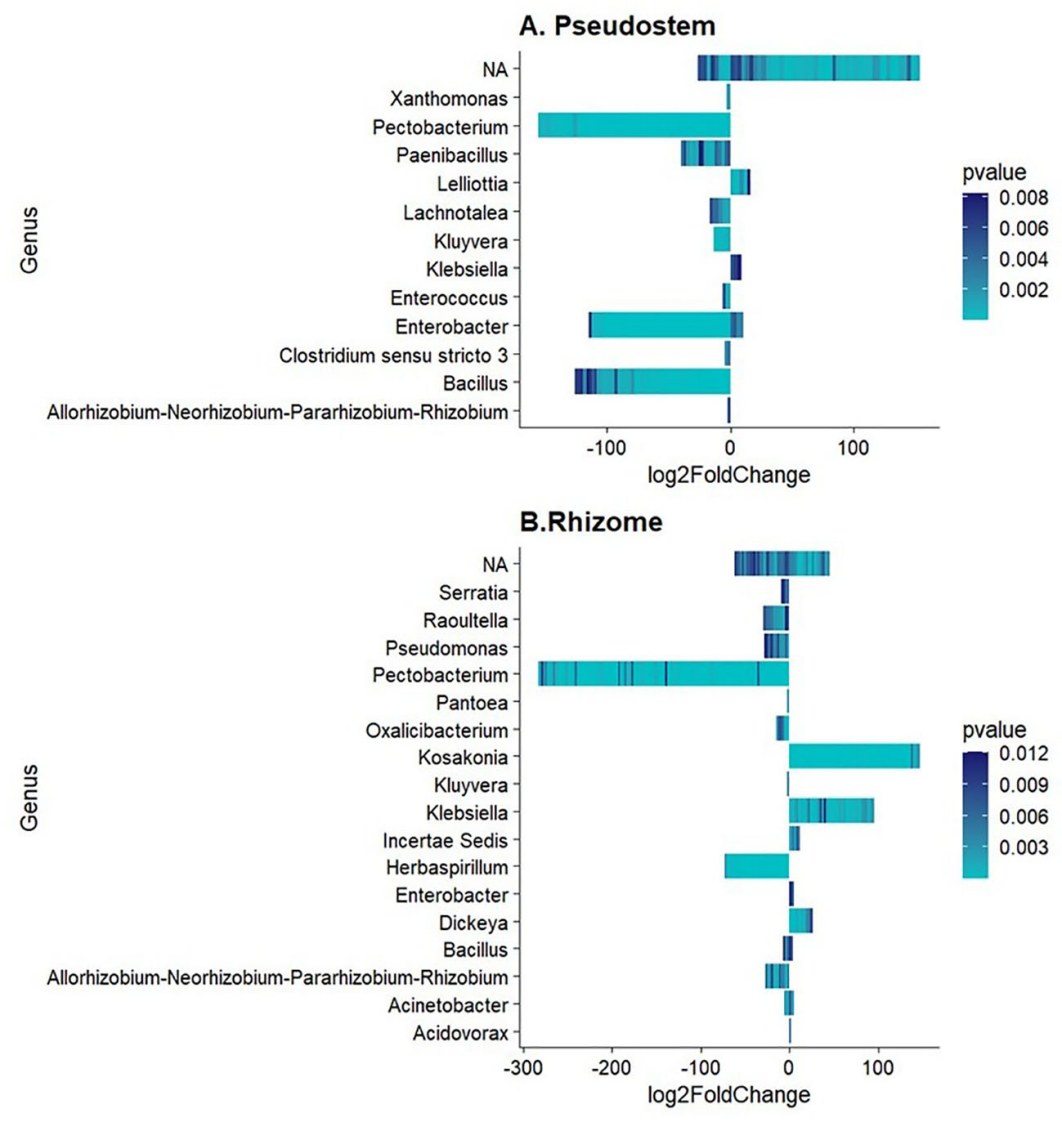
Differentially abundant bacterial genera in **(A)** Pseudostem and **(B)** Rhizome. Log2FoldChange < 0 refers to non-symptomatic samples, while Log2FoldChange > 0 to symptomatic samples.

Differential abundance analysis allowed the identification of several bacterial genera in pseudostem and rhizome, including *Pectobacterium, Bacillus, Enterobacter, Herbaspirillum, Pseudomonas*, and *Paenibacillus* as the genera enriched (p-value < 0.01) in non-symptomatic samples, validating previous results. In contrast, the bacterial genera enriched in symptomatic samples were *Lelliottia, Kosakonia, and Klebsiella* (**Figure 2**). Notably, not significantly enriched or depleted ASVs were found in rhizosphere samples.

**Figure 3:**
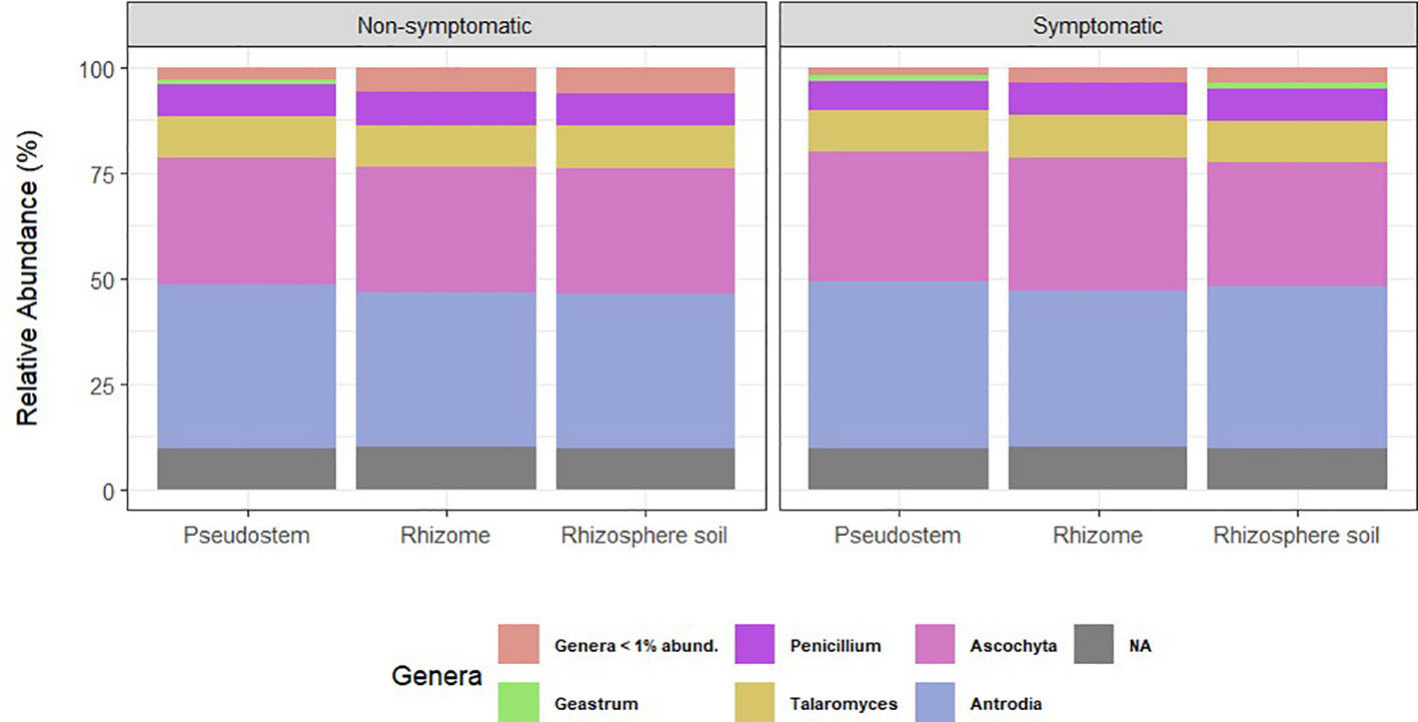
Relative abundance of fungal genera grouped by environment. NA= ASVs with no taxonomic assignment at the genus level. Taxa with abundance < 1% are not shown.

Regarding fungal taxonomic profiles, three samples identified as outliers (0MR217, 1MS211, and 1MS215) were removed from the study. Relative abundance plots were similar for all types of samples, showing that *Ascomycota* and *Basidiomycota* were the dominant phyla in pseudostem, rhizome, and rhizosphere (**Supplementary Figure 4**). The most abundant genera in all sample types were *Antrodia, Ascochyta, Talaromyces*, and *Penicillium* (**Figure 3**).

Heat maps confirmed the dominance of *Antrodia* and *Ascochyta*, followed by *Talaromyces* and *Penicillium* in all the environments tested; other genera present in high proportion were members of the Trichomeriaceae and Ceratobasidiaceae families (**Supplementary Figure 5**).

**Figure 4:**
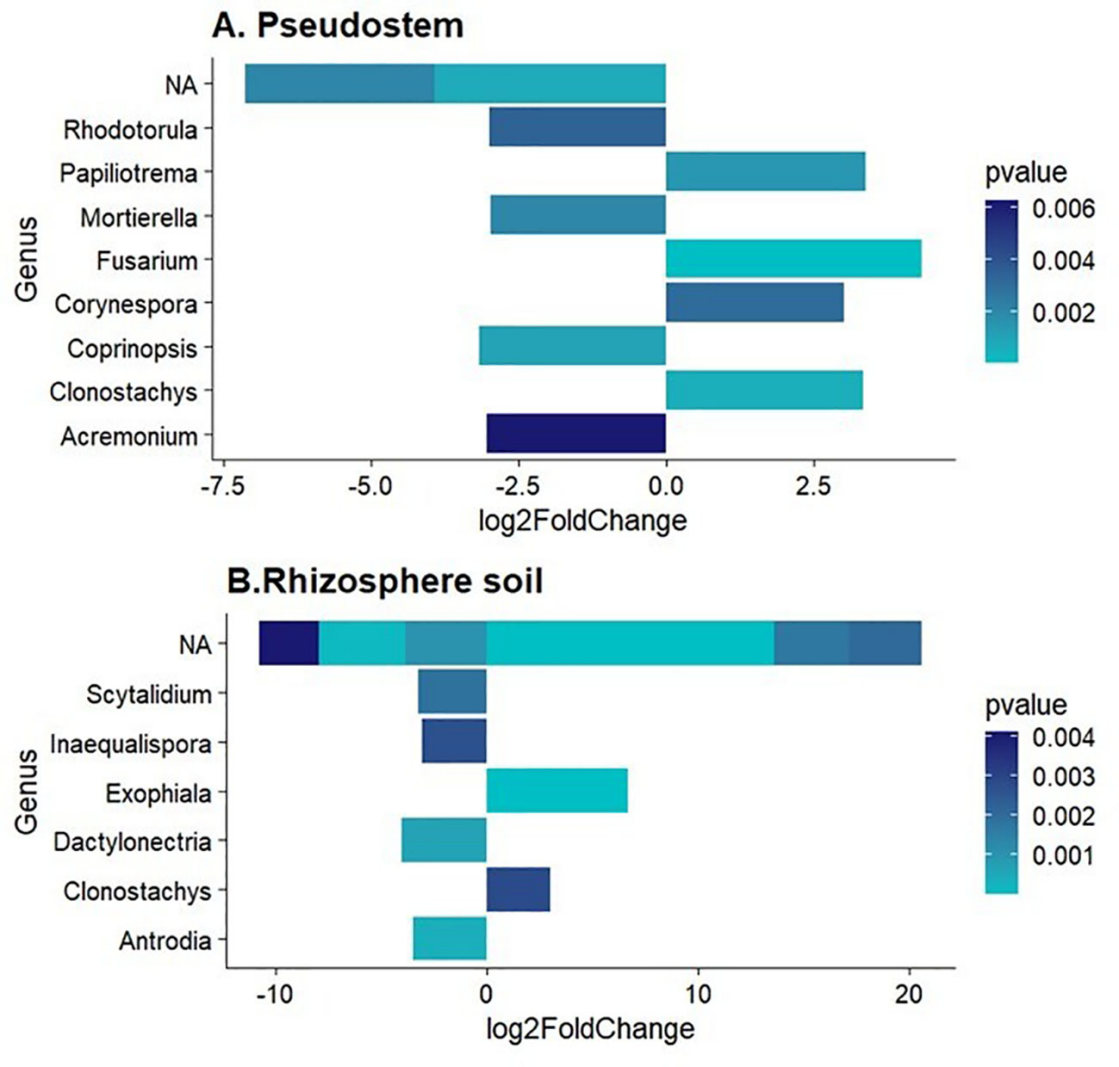
Differentially abundant fungal genera in **(A)** Pseudostem and **(B)** Rhizosphere. Log2FoldChange < 0 refers to non-symptomatic samples, while Log2FoldChange > 0 to symptomatic samples.

The fungal genera enriched (p-value < 0.01) in non-symptomatic pseudostem and rhizosphere were *Rhodorotula. Mortierella, Coprinopsis*, and *Acremonium.* On the other hand, fungal genera enriched in symptomatic samples were *Papilotrema, Fusarium, Corynespora, Clonostachys* and *Exophiala* (**Figure 4**). Notably, not significantly enriched or depleted ASVs were found in rhizome samples.

### 3.2 Microbial ecology analysis

The sequencing depth effort was evaluated using rarefaction curves for both ITS and 16S reads. For both, 16S and ITS datasets, rarefaction curves reached an asymptote, indicating that the sequencing depth covered the taxonomic diversity of the samples (Mbareche et al., 2020). Samples from the rhizosphere had the highest number of ASVs among the 16S samples.

**Figure 5:**
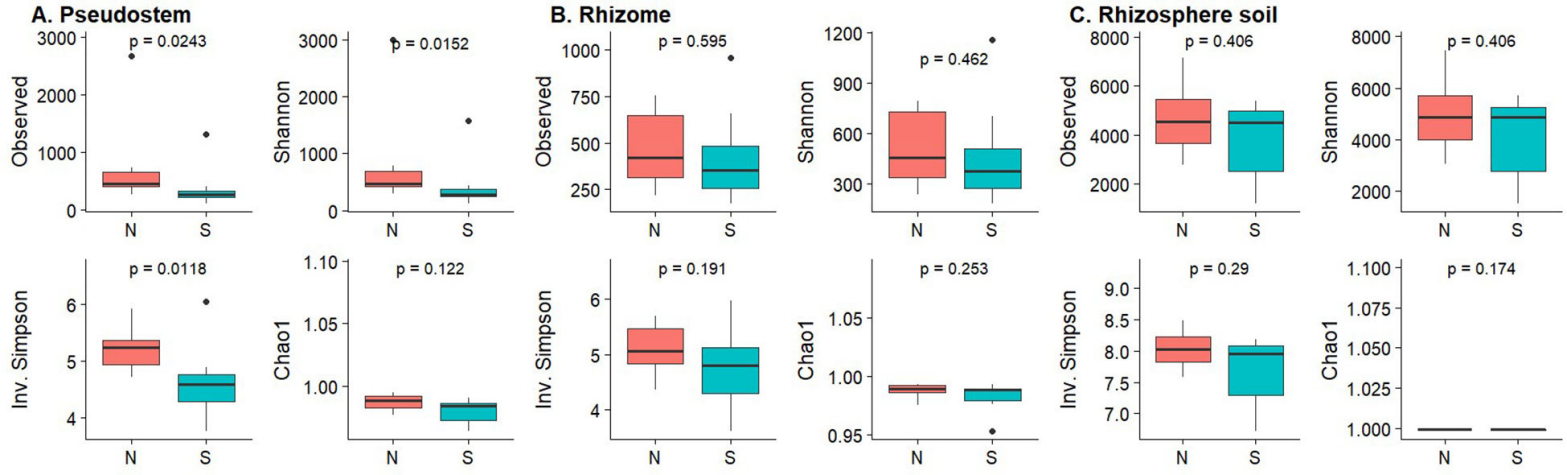
Box plots of alpha diversity measures for bacterial communities based on symptomatology. **(A)** Pseudostem; **(B)** Rhizome; **(C)** Rhizosphere. N, Non-symptomatic; S, Symptomatic. The p-values are indicated at the top of each plot.

For the bacterial community, the rhizosphere samples showed the highest diversity, measured by observed richnness, Shannon index, and Inverse Simpson index. Diversity measures from non-symptomatic samples were higher than those from symptomatic samples (**Figure 5**); however, significant differences were observed only for pseudostem, as determined by the Kruskal-Wallis test (p-value < 0.05) (**Figure 5**). For the fungal community, richness and diversity indices were similar across all samples, and there were no significant differences in the alpha indices regarding symptomatology (**Supplementary Figure 6**).

**Figure 6:**
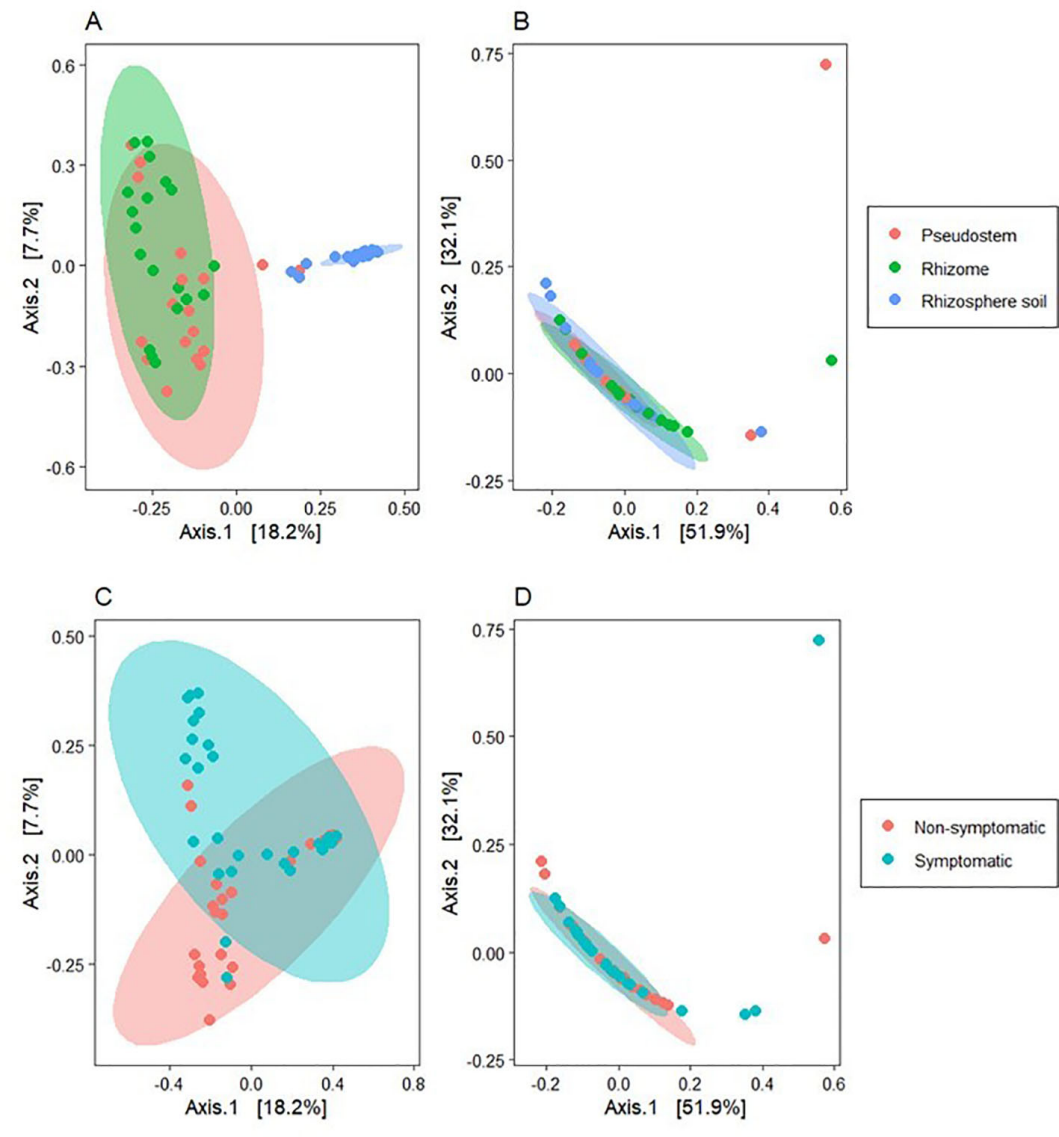
Beta diversity analysis using principal coordinates analysis (PCoA) with the Bray-Curtis distance metric. **(A)** Bacterial communities grouped by environment; **(B)** Fungal communities grouped by environment; **(C)** Bacterial communities grouped by symptomatology; **(D)** Fungal communities grouped by symptomatology. The axes in the figure represent the percentage of the total variation explained by the corresponding principal coordinates.

The PCoA analysis using the Bray-Curtis distances, showed aggrupation in samples based on symptomatology and sample type (**Figure 6**), showing a clear differentiation between rhizosphere and plant tissue samples for bacteria, with significant differences between samples from symptomatic and non-symptomatic plants in all sample types. However, differences in fungal communities were not significant (PERMANOVA; **Table 1**).

**Table 1:** PERMANOVA based on Bray-Curtis distance for the microbial community.

Community	Source of variation	R^2^	F	P-Value
Bacterial	Symptomatology	0.0349	2.5266	0.002 **
	Tissue	0.1874	6.7761	0.001 ***
	Symptomatology: Tissue	0.0417	1.5072	0.025 *
Fungal	Symptomatology	0.0084	0.4685	0.821
	Tissue	0.0436	1.2134	0.273
	Symptomatology: Tissue	0.0608	1.6934	0.087.

Significance codes: 0 ‘***’ 0.001 ‘**’ 0.01 ‘*’ 0.05 ‘.’ 0.1’ ‘ 1.

## 4 Discussion

Tropical Race 4 of Fusarium wilt (FWB) poses a major threat to banana production worldwide and despite ongoing management and containment efforts, the disease continues to spread (Martínez et al., 2023). Among various management strategies, increasing attention is being directed toward understanding and improving soil conditions, including the modulation of microbial activity, due to growing evidence that microbial strains and consortia can contribute meaningfully to integrated disease management programs (Köberl et al., 2017; Kumar et al., 2023; Wang et al., 2024). In Ecuador, Fusarium wilt race 1 was responsible for historic epidemics that devastated plantations of the ‘Gros Michel’ banana variety (Moore et al., 1995). Following these outbreaks, ‘Gros Michel’ was largely replaced by Cavendish cultivars (Ploetz, 2005). However, certain production areas within the country continue to report the persistence of the Fusarium–banana pathosystem above mentioned. These localized scenarios provide a valuable opportunity to understand plant-microbe interactions better, develop microbiome-based management approaches, and guide the selection of promising microbial strains for product development (Siegel-Hertz et al., 2018; Bubici et al., 2019). The presence of beneficial microbes in the rhizosphere can suppress soil-borne pathogens such as Foc, likely through competition for resources, production of antimicrobial compounds, and induction of plant defense responses (Nadiah Jamil et al., 2023). Furthermore, the structure and composition of microbial communities in Fusarium wilt-suppressive soils have been characterized (Jamil et al., 2022) and compared to disease-conducive soils (Lv et al., 2023). In this study, we aimed (1) to compare the composition and diversity of bacterial and fungal communities associated with symptomatic and non-symptomatic ‘Gros Michel’ banana plants growing in fields with a high incidence of FWB; and (2) to identify taxa differentially enriched or depleted between symptomatic and non-symptomatic plants.

Our results showed that FWB symptoms in ‘Gros Michel’ bananas grown in Ecuador were more strongly associated with shifts in bacterial community composition than with changes in fungal communities (**Figure 6**). These findings are congruent with the observations of Kaushal et al. (2020a) who reported that symptomatic bananas had a disrupted root microbiome with a higher abundance of *Flavobacteriales* and decreased levels of beneficial *Proteobacteria* such as *Pseudomonadales*. While their study focused on roots, our work extends these observations to internal plant tissues, particularly in pseudostem tissues, where higher bacterial diversity was observed in non-symptomatic plants (**Figure 5**). This result highlights that microbial shifts are not restricted to the rhizosphere but permeate the banana holobiont.

We found *Gammaproteobacteria* taxa such as *Lelliota, Kosakonia*, and *Klebsiella* differentially abundant in symptomatic tissue, specifically in rhizome and pseudostem. Members of the *Gammaproteobacteria* have been previously found as the dominant endophytic bacteria colonizing roots of symptomatic banana plants of the cultivar Mchare (Kaushal et al., 2020a). However, the symptomatic samples of Mchare were dominated by *Pseudomonales*, *Rhizobiales* and *Burkholderiale*s (Kaushal et al., 2020a). Our findings build on the evidence that *Klebsiella* is a frequently found bacterium in banana tissues (Lian et al., 2008; Andrade et al., 2014; Sekhar et al., 2015; Marcano et al., 2016; Thomas and Sekhar, 2017). In our study, *Klebsiella* was among the most abundant taxa detected in both symptomatic and non-symptomatic rhizome samples. Furthermore, *Klebsiella* spp. and *Enterobacter* spp. (*Enterobacteriaceae*) have been so frequently observed in association with banana that their vertical transmission across plant generations has been proposed (Liu et al., 2019).

On the other hand, *Kosakonia*, another endophytic member of the *Enterobacteriaceae* family, has also drawn attention for its role in shaping the health of banana plants, particularly in the context of FWB. In our study, *Kosakonia* showed significantly higher abundance in symptomatic plant tissues, suggesting a potential opportunistic behavior under disease pressure. However, Liu et al. (2019) proposed a contrasting view, identifying *Kosakonia* as a keystone taxon with beneficial traits for the host plant, including growth promotion and enhanced resistance to Fusarium wilt. These findings suggest that the function of *Kosakonia* within the plant holobiont may be context-dependent, influenced by host status, microbial interactions, and environmental stressors. As for the genus *Lelliottia*, which was also found to be differentially abundant in symptomatic pseudostem samples, its role in banana remains largely uncharacterized. However, its phylogenetic proximity to *Kosakonia* and *Klebsiella*, both members of the *Enterobacteriaceae* family, suggests it may share functional traits such as endophytic colonization and modulation of host defense response (Macedo-Raygoza et al., 2019; Nakkeeran et al., 2021).

By contrast, non-symptomatic tissues exhibited differentially abundant taxa such as *Pectobacterium*, *Bacillus*, *Enterobacter*, *Herbaspirillum*, *Pseudomonas*, and *Paenibacillus*, genera with well-known roles in plant growth promotion and pathogen suppression. Similar findings were reported by Kaushal et al. (2020a), who noted that asymptomatic plants appeared to carry more diverse and beneficial microbial community structures in their tissues. This suggests that healthy plants might actively guard and preserve microorganisms that assist in tolerating diseases. *Bacillus* and *Paenibacillus* are two of the most important microbial members that possess the ability to inhibit pathogens through lipopeptides and antibiotics (Béchet et al., 2012; Cao et al., 2018). These microorganisms are also capable of inducing the plant’s own defense mechanisms (Nadiah Jamil et al., 2023). The presence of these genera in non-symptomatic tissues indicates that they may constitute a defense barrier against Foc and potentially mitigate its effects. Supporting this statement, a study by Cao et al. (2018) presented compelling evidence with *Bacillus velezensis’* antifungal activity against Foc TR4 and *Fusarium oxysporum* f. sp. *cucumerinum* in confrontation assays, where an important inhibition of fungal growth was found. They also tested lipopeptide extracts, with iturin achieving up to ~60% inhibition of Foc TR4 spore germination.

The significant presence of *Pseudomonas* in non-symptomatic tissue samples observed in this study reinforces its proposed role as a beneficial member of the banana microbiome. *Pseudomonas* spp. have been frequently associated with disease-suppressive environments, widely recognized for their ability to effectively colonize plant tissues, promote plant growth, produce siderophores, suppress phytopathogens, and induce systemic resistance responses (Zhou et al., 2019; Wong et al., 2021; Lv et al., 2023). For instance, Shen et al. (2015) identified *Pseudomonas* as one of the most enriched genera in soils suppressive to FWB, while Köberl et al. (2017) found it more abundant in non-symptomatic plants, alongside other potentially protective *Gammaproteobacteria*. Similarly, Kaushal et al. (2020b) observed a high colonization of *Pseudomonadales* in the roots of asymptomatic plants in two banana varieties, highlighting its consistent association with healthier phenotypes.

Taken together, these findings suggest that certain microbial taxa may serve as indicators of plant health, and that their presence in non-symptomatic tissues is unlikely to be random. Instead, they may constitute part of a stable and protective microbiome that contributes to disease resistance. This notion aligns with several studies that describe the existence of “core microbiomes” in banana plants, typically composed of genera such as *Bacillus, Pseudomonas*, and *Paenibacillus*, which have been associated with both plant growth promotion and biocontrol functions (Massart et al., 2015; Cao et al., 2018; Wong et al., 2021; Gómez-Lama Cabanás et al., 2022; Lv et al., 2023).

In our study, the genus *Enterobacter* was differentially abundant in non-symptomatic pseudostem and rhizome tissues, suggesting a potential endophytic role under balanced microbial conditions. This observation contrasts with findings reported by Köberl et al. (2017), who documented an increased abundance of *Enterobacter* and other *Enterobacteriaceae*, such as *Erwinia*, in banana plants exhibiting FWB symptoms, particularly in above-ground tissues from Costa Rican farms. Such differences highlight the ecological plasticity of *Enterobacter*, which includes both beneficial and opportunistic strains. Its presence in non-symptomatic tissues may reflect a mutualistic interaction that supports plant health, for instance, through nutrient mobilization or modulation of plant defenses (Macedo-Raygoza et al., 2019). However, its recurrent enrichment in symptomatic plants across multiple studies raises the possibility of facultative opportunistic behavior, where certain strains may exploit weakened plant tissues or altered host environments caused by pathogen invasion.

The presence of *Pectobacterium* in non-symptomatic tissues is unexpected, given its well-established role as a causative agent of soft rot diseases in various crops (Davidsson et al., 2013). However, in our study, no signs of rot or tissue degradation were observed. This finding aligns with the results of Birt et al. (2022), who identified *Pectobacterium* as part of the core bacterial microbiome of banana (*Musa* spp.), consistently present across diverse genotypes and geographical locations. Such ubiquity suggests that certain *Pectobacterium* strains may exist as endophytes within banana plants without eliciting disease symptoms, potentially due to host tolerance or microbial community interactions that suppress pathogenicity. The consistent detection of *Pectobacterium* in healthy tissues raises questions about its ecological role, whether it functions as a mutualist, a latent pathogen, or a neutral inhabitant within the banana microbiome. These contrasting patterns underscore the necessity for strain-level characterization to determine whether specific *Pectobacterium* and *Enterobacter* strains contribute to disease suppression, persist as neutral endophytes, or enhance plant fitness (Birt et al., 2022). However, their consistent association with non-symptomatic tissues supports the need for further research. Future studies should test whether these bacteria can suppress Foc in controlled experiments and understand how they interact with the plant and other microbes. If confirmed, these genera could be used as biological indicators or even as biocontrol agents in banana disease management programs.

*Herbaspirillum* has also been reported as a beneficial endophyte in bananas and other crops. This bacterial genus is known for enhancing nutrient availability, improving stress tolerance, and contributing to disease resistance in host plants (Weber et al., 2007; Das et al., 2024). Previous research has demonstrated that diazotrophic endophytes, including *Burkholderia* and *Herbaspirillum* spp., can suppress Foc propagules and promote plant growth, supporting their potential as biological control agents against Foc TR4 (Weber et al., 2007).

It is important to note that our results do not demonstrate a direct curative effect of these bacteria against FWB. However, recent studies have shown that microbial biocontrol agents, particularly those based on *Bacillus* spp., can significantly reduce disease incidence and alleviate physiological damage in banana plants affected by Foc (Izquierdo-García et al., 2024). While not a definitive solution, such microbial strains represent promising complementary tools within an integrated disease management framework, particularly by delaying pathogen colonization and enhancing plant physiological resilience.

In contrast to the bacterial communities, fungal communities associated with ‘Gros Michel’ banana plants did not exhibit significant differences in alpha diversity or richness across tissue types or symptom categories. Ascomycota and Basidiomycota were the dominant fungal phyla in samples, including pseudostem, rhizome, and rhizosphere, regardless of plant health status. This observation aligns with previous studies that identified these phyla as core members of the banana-associated mycobiome in monoculture systems (Shen et al., 2017).

Nonetheless, differential abundance analysis revealed specific genera enriched in asymptomatic plants, including *Rhodotorula, Mortierella, Coprinopsis*, and *Acremonium*, whereas *Fusarium, Papiliotrema, Corynespora, Clonostachys*, and *Exophiala* were more abundant in symptomatic tissues. The higher abundance of *Fusarium* in diseased plants confirms its role as the causal agent of FWB. Conversely, genera such as *Rhodotorula* and *Mortierella* in non-symptomatic samples may indicate potential protective effects or reflect non-pathogenic opportunistic colonization under healthy conditions. *Mortierella* has been identified as a dominant genus in disease-free banana soils, suggesting its potential role in suppressing FWB through mechanisms such as competition or modulation of the soil microbiome. Similarly, *Acremonium* species, traditionally known for their antibiotic production, have been reported to exhibit antagonistic activity against plant pathogens, indicating their potential as biocontrol agents (Zhou et al., 2019). Although no promising fungal strains were identified in this study, it is noteworthy that several species have been reported with antagonistic activity against Foc (Wong et al., 2021; Izquierdo-García et al., 2024; Jin et al., 2024).

Despite these observations, PERMANOVA analysis did not detect statistically significant differences in fungal community composition based on symptomatology or tissue type. This suggests that fungal community structure is less responsive to disease status than bacterial communities in this pathosystem, and that disease progression may be driven more by the functional behavior of specific pathogenic taxa, particularly *Fusarium*, than by broad shifts in fungal assemblages. This perspective aligns with previous studies. For instance, Kaushal et al. (2020b) observed that bacterial communities exhibited more pronounced shifts in response to FWB than fungal communities, suggesting a stronger association between bacterial community dynamics and disease status. Similarly, Zhou et al. (2019) reported that while *Fusarium* abundance increased in diseased soils, overall fungal community composition did not significantly differ between healthy and diseased plants, highlighting the role of specific pathogenic taxa over broad community changes. These findings support the view that bacterial community composition may serve as a more reliable indicator of plant health and disease progression in the context of FWB.

Although this study provides valuable insights into the association between microbiome structure and FWB symptoms in ‘Gros Michel’ bananas, certain limitations must be acknowledged. The cross-sectional nature of sampling prevents establishing a direct causal relationship between microbial shifts and disease suppression.

In this study, we employed amplicon-based DNA sequencing. This method does not distinguish between DNA from viable and non-viable organisms, potentially leading to an overestimation of certain taxa that are no longer functionally active in the sampled tissues or rhizosphere. Consequently, the taxonomic profiles generated reflect the potential, rather than the active, microbial community composition. This limitation has been acknowledged in previous research, where DNA-based sequencing methods were found to over represent inactive or dead microbial populations, thus not accurately reflecting the metabolically active community members (Jiang et al., 2016).

Future studies should incorporate complementary approaches such as RNA-based sequencing (metatranscriptomics), metabolomics, or culturing techniques. Metatranscriptomics, for instance, allows for the assessment of gene expression profiles, providing insights into the functional activities of microbial communities. Metabolomics can reveal the metabolic profiles and interactions between microbes and host plants, offering a deeper understanding of the functional roles of specific taxa (Ye et al., 2022).

We propose the functional validation of candidate microbial taxa identified in this study (e.g., *Pseudomonas, Paenibacillus, Bacillus, Herbaspirillum*) through controlled bioassays to assess their biocontrol potential against Foc TR4. This aligns with previous efforts by Du et al. (2024) who demonstrated that combining *Paenibacillus polymyxa*, *Trichoderma harzianum*, and carbendazim achieved up to 60.53% control efficiency on disease control in field trials.

Furthermore, the efficacy of these beneficial strains should be evaluated across different *Musa* cultivars, including Cavendish banana and plantain, to determine whether their protective effects are consistent. This is particularly relevant evidence given that both environmental conditions and host genotype can influence the effectiveness of biocontrol agents, as stated by Birt et al. (2022).

Another important consideration is the modulation of microbiomes through the application of organic amendments, a common practice among producers. Therefore, investigating how the incorporation of organic amendments into agricultural systems might enhance the diversity and abundance of beneficial microbial taxa for managing FWB should be a research priority. Previous studies have shown that such practices can significantly reshape microbial community composition and may promote the proliferation of protective microbes (Fu et al., 2017; Hartman et al., 2018). For instance, Xue et al. (2015) find that the use of organic fertilizers has been linked to increased microbial biomass and activity in the banana rhizosphere, contributing to disease suppression.

Future research should also include longitudinal monitoring of microbiome dynamics throughout disease progression and across diverse agroecological settings, including comparisons between organic and conventional production systems. Implementing field-level biosecurity measures and integrating microbiome-based interventions into conventional management strategies may offer a promising avenue for sustainable control of Fusarium wilt in banana production systems.

## Data Availability

The datasets generated in this study are available in the NCBI Sequence Read Archive (SRA) under BioProject accession number PRJNA1218678 [https://www.ncbi.nlm.nih.gov/sra/PRJNA1218678].

## References

[B1] Altendorf S. (2019). Banana Fusarium Wilt Tropical Race 4: A mounting threat to global banana markets? FAO Food Outlook. 12–20. doi: 10.1787/agr_outlook-2019-en

[B2] Anders S. Huber W. (2010). Differential expression analysis for sequence count data. Genome Biol. 11, 1–12. doi: 10.1186/GB-2010-11-10-R106/COMMENTS PMC3218662 20979621

[B3] Andersen K. S. Kirkegaard R. H. Karst S. M. Albertsen M. (2018). ampvis2: an R package to analyse and visualise 16S rRNA amplicon data. bioRxiv, 299537. doi: 10.1101/299537

[B4] Andrade L. F. de Souza G. L. O. D. Nietsche S. Xavier A. A. Costa M. R. Cardoso A. M. S.. (2014). Analysis of the abilities of endophytic bacteria associated with banana tree roots to promote plant growth. J. Microbiol. 52, 27–34. doi: 10.1007/S12275-014-3019-2 24390835

[B5] Andrews S. (2019). Babraham Bioinformatics - FastQC A Quality Control tool for High Throughput Sequence Data. Available online at: https://www.bioinformatics.babraham.ac.uk/projects/fastqc/ (Accessed January 9, 2022).

[B6] Béchet M. Caradec T. Hussein W. Abderrahmani A. Chollet M. Leclére V.. (2012). Structure, biosynthesis, and properties of kurstakins, nonribosomal lipopeptides from Bacillus spp. Appl. Microbiol. Biotechnol. 95, 593–600. doi: 10.1007/S00253-012-4181-2/METRICS 22678024

[B7] Benitez M. S. Malacrino A. Jelmer P. (2021). Microbiome metabarcoding workshop (The Ohio State University). Available at: https://mcic-osu.github.io/2020-12-microbiomics-workshop/index.html.

[B8] Berg G. Rybakova D. Grube M. Köberl M. (2016). The plant microbiome explored: implications for experimental botany. J. Exp. Bot. 67, 995–1002. doi: 10.1093/JXB/ERV466 26547794 PMC5395086

[B9] Birt H. W. G. Pattison A. B. Skarshewski A. Daniells J. Raghavendra A. Dennis P. G. (2022). The core bacterial microbiome of banana (Musa spp.). Environ. Microbiome 17, 1–19. doi: 10.1186/S40793-022-00442-0 36076285 PMC9461194

[B10] Bolger A. M. Lohse M. Usadel B. (2014). Trimmomatic: a flexible trimmer for Illumina sequence data. Bioinformatics 30, 2114–2120. doi: 10.1093/BIOINFORMATICS/BTU170 24695404 PMC4103590

[B11] Bubici G. Kaushal M. Prigigallo M. I. Cabanás C. G. L. Mercado-Blanco J. (2019). Biological control agents against Fusarium wilt of banana. Front. Microbiol 10. doi: 10.3389/fmicb.2019.00616 PMC6459961 31024469

[B12] Callahan B. McMurdie P. Rosen M. Han A. Johnson A. Holmes S. (2016). DADA2: High-resolution sample inference from Illumina amplicon data. Nat. Methods 13, 581–583. doi: 10.1038/NMETH.3869 27214047 PMC4927377

[B13] Callahan B. Wong J. Heiner C. Oh S. Theriot C. M. Gulati A. S.. (2019). High-throughput amplicon sequencing of the full-length 16S rRNA gene with single-nucleotide resolution. Nucleic Acids Res. 47, e103–e103. doi: 10.1093/NAR/GKZ569 31269198 PMC6765137

[B14] Callens K. Fontaine F. Sanz Y. Bogdanski A. D’Hondt K. Lange L.. (2022). Microbiome-based solutions to address new and existing threats to food security, nutrition, health and agrifood systems’ sustainability. Front. Sustain Food Syst. 6. doi: 10.3389/FSUFS.2022.1047765/BIBTEX

[B15] Cao Y. Pi H. Chandrangsu P. Li Y. Wang Y. Zhou H.. (2018). Antagonism of Two Plant-Growth Promoting Bacillus velezensis Isolates Against Ralstonia solanacearum and Fusarium oxysporum. Sci. Rep. 8. doi: 10.1038/S41598-018-22782-Z PMC5847583 29531357

[B16] Cha J. Y. Han S. Hong H. J. Cho H. Kim D. Kwon Y.. (2016). Microbial and biochemical basis of a Fusarium wilt-suppressive soil. ISME J. 10, 119–129. doi: 10.1038/ISMEJ.2015.95 26057845 PMC4681868

[B17] Chittarath K. Nguyen C. H. Bailey W. C. Zheng S. J. Mostert D. Viljoen A.. (2022). Geographical distribution and genetic diversity of the banana fusarium wilt fungus in Laos and Vietnam. J. Fungi 8, 46. doi: 10.3390/JOF8010046/S1 PMC8781582 35049986

[B18] Das D. Baro T. Brahma S. Saikia D. Das S. (2024). “ENDOPHYTIC BACTERIA AND THEIR POTENTIAL APPLICATIONS IN AGRICULTURE,” in Futuristic trends in agriculture engineering & Food sciences, vol. 3. , 23–30. Book 5. Iterative International Publisher (IIP) Selfypage Developers Pvt Ltd. doi: 10.58532/V3BCAG5P1CH2

[B19] Davidsson P. R. Kariola T. Niemi O. Tapio Palva E. (2013). Pathogenicity of and plant immunity to soft rot pectobacteria. Front. Plant Sci. 4. doi: 10.3389/FPLS.2013.00191/XML/NLM PMC3678301 23781227

[B20] de Figueiredo F. Kaplan S. Magdama F. A. Potts M. D. Espinel R. L. Zilberman D. (2023). Estimating worldwide benefits from improved bananas resistant to Fusarium Wilt Tropical race 4. J. Agric. Appl. Economics Assoc. 2, 20–34. doi: 10.1002/JAA2.41

[B21] Dixon P. (2003). VEGAN, A package of R functions for community ecology on JSTOR. J. Gegetation Sci. 14, 927–930.

[B22] Du C. Yang D. Jiang S. Zhang J. Ye Y. Pan L.. (2024). Biocontrol agents inhibit banana fusarium wilt and alter the rooted soil bacterial community in the field. J. Fungi 10. doi: 10.3390/JOF10110771 PMC11595440 39590690

[B23] Food and Agriculture Organization of the United Nations (2023). FAOSTAT. Available online at: https://www.fao.org/faostat/en/data/QI (Accessed February 2, 2023).

[B24] Foster Z. S. L. Sharpton T. J. Grünwald N. J. (2017). Metacoder: An R package for visualization and manipulation of community taxonomic diversity data. PloS Comput. Biol. 13, e1005404. doi: 10.1371/JOURNAL.PCBI.1005404 28222096 PMC5340466

[B25] Fu L. Penton C. R. Ruan Y. Shen Z. Xue C. Li R.. (2017). Inducing the rhizosphere microbiome by biofertilizer application to suppress banana Fusarium wilt disease. Soil Biol. Biochem. 104, 39–48. doi: 10.1016/J.SOILBIO.2016.10.008

[B26] Garcia R. O. Rivera-Vargas L. I. Ploetz R. Correll J. C. Irish B. M. (2018). Characterization of Fusarium spp. isolates recovered from bananas (Musa spp.) affected by Fusarium wilt in Puerto Rico. Eur. J. Plant Pathol. 152, 599–611. doi: 10.1007/S10658-018-1503-Y/FIGURES/4

[B27] García-Bastidas F. A. Arango-Isaza R. Rodriguez-Cabal H. A. Seidl M. F. Cappadona G. Segura R.. (2022). Induced resistance to Fusarium wilt of banana caused by Tropical Race 4 in Cavendish cv Grand Naine bananas after challenging with avirulent Fusarium spp. PloS One 17, e0273335. doi: 10.1371/JOURNAL.PONE.0273335 36129882 PMC9491598

[B28] Gómez-Lama Cabanás C. Wentzien N. M. Zorrilla-Fontanesi Y. Valverde-Corredor A. Fernández-González A. J. Fernández-López M.. (2022). Impacts of the biocontrol strain pseudomonas simiae PICF7 on the banana holobiont: alteration of root microbial co-occurrence networks and effect on host defense responses. Front. Microbiol 13. doi: 10.3389/FMICB.2022.809126/FULL PMC8885582 35242117

[B29] Hartman K. van der Heijden M. G. A. Wittwer R. A. Banerjee S. Walser J. C. Schlaeppi K. (2018). Cropping practices manipulate abundance patterns of root and soil microbiome members paving the way to smart farming. Microbiome 6, 1–14. doi: 10.1186/S40168-017-0389-9/FIGURES/5 29338764 PMC5771023

[B30] Herlemann D. P. R. Labrenz M. Jürgens K. Bertilsson S. Waniek J. J. Andersson A. F. (2011). Transitions in bacterial communities along the 2000 km salinity gradient of the Baltic Sea. ISME J. 5, 1571–1579. doi: 10.1038/ismej.2011.41 21472016 PMC3176514

[B31] Hou B. H. Tsai Y. H. Chiang M. H. Tsao S. M. Huang S. H. Chao C. P.. (2022). Cultivar-specific markers, mutations, and chimerisim of Cavendish banana somaclonal variants resistant to Fusarium oxysporum f. sp. cubense tropical race 4. BMC Genomics 23. doi: 10.1186/s12864-022-08692-5 PMC9233791 35752751

[B32] Illumina (2018). 16S metagenomic sequencing library preparation preparing 16S ribosomal RNA gene amplicons for the illumina miSeq system. Available online at: https://support.illumina.com/documents/documentation/chemistry_documentation/16s/16s-metagenomic-library-prep-guide-15044223-b.pdf (Accessed March 11, 2021).

[B33] Illumina (2019). “Fungal metagenomic sequencing demonstrated protocol,” in Preparing ITS amplicons for sequencing on illumina sequencing systems. Available at: https://support.illumina.com/content/dam/illumina-support/documents/documentation/chemistry_documentation/metagenomic/fungal-metagenomic-demonstrated-protocol-1000000064940-01.pdf.

[B34] Illumina (2021). NGS Workflow Steps | Illumina sequencing workflow. Illumina Support Center. Available online at: https://www.illumina.com/science/technology/next-generation-sequencing/beginners/ngs-workflow.html (Accessed July 14, 2021).

[B35] Izquierdo-García L. F. Carmona-Gutiérrez S. L. Moreno-Velandia C. A. Villarreal-Navarrete A.d. P. Burbano-David D. M. Quiroga-Mateus R. Y.. (2024). Microbial-Based Biofungicides Mitigate the Damage Caused by Fusarium oxysporum f. sp. cubense Race 1 and Improve the Physiological Performance in Banana. J. Fungi 10, 419. doi: 10.3390/JOF10060419/S1 PMC11204473 38921405

[B36] Jamil F. N. Hashim A. M. Yusof M. T. Saidi N. B. (2022). Analysis of soil bacterial communities and physicochemical properties associated with Fusarium wilt disease of banana in Malaysia. Sci. Rep. 12, 1–11. doi: 10.1038/S41598-022-04886-9;SUBJMETA=2169,2565,326,449,631;KWRD=MICROBIAL+COMMUNITIES,MICROBIOLOGY,PLANT+IMMUNITY 35046475 PMC8770495

[B37] Jiang Y. Xiong X. Danska J. Parkinson J. (2016). Metatranscriptomic analysis of diverse microbial communities reveals core metabolic pathways and microbiomespecific functionality. Microbiome 4, 1–18. doi: 10.1186/S40168-015-0146-X/FIGURES/5 26757703 PMC4710996

[B38] Jin L. Huang R. Zhang J. Li Z. Li R. Li Y.. (2024). Identification and Characterization of Endophytic Fungus DJE2023 Isolated from Banana (Musa sp. cv. Dajiao) with Potential for Biocontrol of Banana Fusarium Wilt. J. Fungi 10, 877. doi: 10.3390/JOF10120877/S1 PMC11677757 39728374

[B39] Jones D. R. (2019). Handbook of diseases of banana, abaca and enset (CABI Digital Library). doi: 10.1079/9781780647197.0000

[B40] Kaushal M. Mahuku G. Swennen R. (2020a). Metagenomic insights of the root colonizing microbiome associated with symptomatic and non-symptomatic bananas in Fusarium wilt infected fields. Plants 9. doi: 10.3390/plants9020263 PMC7076721 32085593

[B41] Kaushal M. Swennen R. Mahuku G. (2020b). Unlocking the Microbiome Communities of Banana (Musa spp.) under Disease Stressed (Fusarium wilt) and Non-Stressed Conditions. Microorganisms 8, 443. doi: 10.3390/MICROORGANISMS8030443 32245146 PMC7144012

[B42] Köberl M. Dita M. Martinuz A. Staver C. Berg G. (2017). Members of Gammaproteobacteria as indicator species of healthy banana plants on Fusarium wilt-infested fields in Central America. Sci. Rep. 7. doi: 10.1038/srep45318 PMC5366900 28345666

[B43] Kumar U. Sheleke R. M. Singh R. (2023). Editorial: Soil-plant-microbe interactions: An innovative approach towards improving soil health and plant growth. Front. Agron. 5. doi: 10.3389/FAGRO.2023.1165328/BIBTEX

[B44] Kuźniar A. Włodarczyk K. Grządziel J. Goraj W. Gałązka A. Wolińska A. (2020). Culture-independent analysis of an endophytic core microbiome in two species of wheat: Triticum aestivum L. (cv. ‘Hondia’) and the first report of microbiota in Triticum spelta L. (cv. ‘Rokosz’). Syst. Appl. Microbiol 43, 126025. doi: 10.1016/J.SYAPM.2019.126025 31704194

[B45] Lian J. Wang Z. Zhou S. (2008). Response of endophytic bacterial communities in banana tissue culture plantlets to Fusarium wilt pathogen infection. J. Gen. Appl. Microbiol. 54, 83–92. doi: 10.2323/JGAM.54.83 18497482

[B46] Liu Y. Zhu A. Tan H. Cao L. Zhang R. (2019). Engineering banana endosphere microbiome to improve Fusarium wilt resistance in banana. Microbiome 7, 1–15. doi: 10.1186/s40168-019-0690-x 31092296 PMC6521393

[B47] Luz M. (2019). Statistical analysis of metagenomics data. Genomics Inform 17. doi: 10.5808/GI.2019.17.1.e6 PMC6459172 30929407

[B48] Lv N. Tao C. Ou Y. Wang J. Deng X. Liu H.. (2023). Root-Associated Antagonistic Pseudomonas spp. Contribute to Soil Suppressiveness against Banana Fusarium Wilt Disease of Banana. Microbiol Spectr. 11. doi: 10.1128/SPECTRUM.03525-22 PMC10100972 36786644

[B49] Macedo-Raygoza G. M. Valdez-Salas B. Prado F. M. Prieto K. R. Yamaguchi L. F. Kato M. J.. (2019). Enterobacter cloacae, an endophyte that establishes a nutrient-transfer symbiosis with banana plants and protects against the black sigatoka pathogen. Front. Microbiol 10. doi: 10.3389/FMICB.2019.00804 PMC6513882 31133991

[B50] Magdama F. Monserrate-Maggi L. Serrano L. García Onofre J. Jiménez-Gasco M.d. M. (2020). Genetic Diversity of Fusarium oxysporum f. sp. cubense, the Fusarium Wilt Pathogen of Banana, in Ecuador. Plants 9, 1133. doi: 10.3390/plants9091133 32882937 PMC7570379

[B51] Magdama Tobar F. A. (2017). Population biology of Fusarium oxysporum associated with banana in Ecuador (State College: The Pennsylvania State University).

[B52] Marcano I. E. Díaz-Alcántara C. A. Urbano B. González-Andrés F. (2016). Assessment of bacterial populations associated with banana tree roots and development of successful plant probiotics for banana crop. Soil Biol. Biochem. 99, 1–20. doi: 10.1016/J.SOILBIO.2016.04.013

[B53] Martínez G. Olivares B. O. Rey J. C. Rojas J. Cardenas J. Muentes C.. (2023). The advance of fusarium wilt tropical race 4 in musaceae of latin america and the caribbean: current situation. Pathogens 12, 277. doi: 10.3390/PATHOGENS12020277 36839549 PMC9963102

[B54] Massart S. Perazzolli M. Höfte M. Pertot I. Jijakli M. H. (2015). Impact of the omic technologies for understanding the modes of action of biological control agents against plant pathogens. BioControl 60, 725–746. doi: 10.1007/S10526-015-9686-Z

[B55] Mbareche H. Dumont-Leblond N. Bilodeau G. J. Duchaine C. (2020). An overview of bioinformatics tools for DNA meta-barcoding analysis of microbial communities of bioaerosols: digest for microbiologists. Life 10, 1–20. doi: 10.3390/LIFE10090185 PMC7555798 32911871

[B56] McLaren M. R. (2020). Silva SSU taxonomic training data formatted for DADA2 (Silva version 138). doi: 10.5281/ZENODO.3986799

[B57] McMurdie P. J. Holmes S. (2013). phyloseq: an R package for reproducible interactive analysis and graphics of microbiome census data. PloS One 8, e61217. doi: 10.1371/JOURNAL.PONE.0061217 23630581 PMC3632530

[B58] Mejías R. Hernández Y. Magdama F. Mostert D. Bothma S. Paredes E. M.. (2023). First Report of Fusarium Wilt of Cavendish Bananas Caused by Fusarium oxysporum f. sp. cubense Tropical Race 4 in Venezuela. J. Plant Dis. 107, 3297. doi: 10.1094/PDIS-04-23-0781-PDN

[B59] Mitter B. Brader G. Pfaffenbichler N. Sessitsch A. (2019). Next generation microbiome applications for crop production — limitations and the need of knowledge-based solutions. Curr. Opin. Microbiol 49, 59–65. doi: 10.1016/J.MIB.2019.10.006 31731227

[B60] Moore N. Bentley S. Pegg K. Jones D. (1995). Fusarium wilt of banana. doi: 10.5555/19971000686

[B61] Nadiah Jamil F. Mohd Hashim A. Termizi Yusof M. Baity Saidi N. (2023). Association of soil fungal community composition with incidence of Fusarium wilt of banana in Malaysia. Mycologia. 115, 178–186. doi: 10.1080/00275514.2023.2180975 36893072

[B62] Nakkeeran S. Rajamanickam S. Saravanan R. Vanthana M. Soorianathasundaram K. (2021). Bacterial endophytome-mediated resistance in banana for the management of Fusarium wilt. 3 Biotech. 11, 267. doi: 10.1007/S13205-021-02833-5 PMC8124033 34017673

[B63] Nilsson R. H. Larsson K. H. Taylor A. F. S. Bengtsson-Palme J. Jeppesen T. S. Schigel D.. (2019). The UNITE database for molecular identification of fungi: handling dark taxa and parallel taxonomic classifications. Nucleic Acids Res. 47, D259–D264. doi: 10.1093/NAR/GKY1022 30371820 PMC6324048

[B64] Ordonez N. Seidl M. F. Waalwijk C. Drenth A. Kilian A. Thomma B. P. H. J.. (2015). Worse comes to worst: bananas and Panama disease—When plant and pathogen clones meet. PloS Pathog 11, e1005197. doi: 10.1371/JOURNAL.PPAT.1005197 26584184 PMC4652896

[B65] Pegg K. G. Coates L. M. O’Neill W. T. Turner D. W. (2019). The epidemiology of fusarium wilt of banana. Front. Plant Sci. 0. doi: 10.3389/FPLS.2019.01395 PMC6933004 31921221

[B66] Ploetz R. C. (2005). Panama disease: an old nemesis rears its ugly head: part 1. The beginnings of the banana export trades. Plant Health Prog. 6, 18. doi: 10.1094/PHP-2005-1221-01-RV

[B67] Ploetz R. C. Evans E. A. (2015). The future of global banana production. Hortic. Rev. (Am Soc. Hortic. Sci) 43, 311–351. doi: 10.1002/9781119107781.CH06

[B68] Ploetz R. C. Kema G. H. J. Ma L. J. (2015). Impact of diseases on export and smallholder production of banana. Annu. Rev. Phytopathol. 53, 269–288. doi: 10.1146/ANNUREV-PHYTO-080614-120305 26002290

[B69] Rausch P. Rühlemann M. Hermes B. M. Doms S. Dagan T. Dierking K.. (2019). Comparative analysis of amplicon and metagenomic sequencing methods reveals key features in the evolution of animal metaorganisms. Microbiome 7, 1–19. doi: 10.1186/S40168-019-0743-1 31521200 PMC6744666

[B70] R Core Development Team (2015). R: a language and environment for statistical computing, 3.2.1. R Foundation for Statistical Computing. doi: 10.1017/CBO9781107415324.004

[B71] Roberts J. M. Carvalhais L. C. O’Dwyer C. Rincón-Flórez V. A. Drenth A. (2024). Diagnostics of Fusarium wilt in banana: Current status and challenges. Plant Pathol. 73, 760–776. doi: 10.1111/PPA.13863

[B72] Sahu S. K. Thangaraj M. Kathiresan K. (2012). DNA extraction protocol for plants with high levels of secondary metabolites and polysaccharides without using liquid nitrogen and phenol. ISRN Mol. Biol. 2012, 1–6. doi: 10.5402/2012/205049 PMC4890884 27335662

[B73] Schlaeppi K. Bulgarelli D. (2015). The plant microbiome at work 28. J. Mol. Plant-Microbe Interactions. 212–217. doi: 10.1094/MPMI-10-14-0334-FI 25514681

[B74] Schliep K. P. (2011). phangorn: phylogenetic analysis in R. Bioinformatics 27, 592–593. doi: 10.1093/BIOINFORMATICS/BTQ706 21169378 PMC3035803

[B75] Sekhar A. C. Thomas P. Sekhar A. C. Thomas P. (2015). Isolation and Identification of Shoot-Tip Associated Endophytic Bacteria from Banana cv. Grand Naine and Testing for Antagonistic Activity against Fusarium oxysporum f. sp. cubense. Am. J. Plant Sci. 6, 943–954. doi: 10.4236/AJPS.2015.67101

[B76] Shen Z. Penton C. Lv R. Xue C. Yuan X. Ruan Y.. (2017). Banana fusarium wilt disease incidence is influenced by shifts of soil microbial communities under different monoculture spans. Microb Ecol. 75, 739–750. doi: 10.1007/S00248-017-1052-5 28791467

[B77] Shen Z. Ruan Y. Xue C. Zhong S. Li R. Shen Q. (2015). Soils naturally suppressive to banana Fusarium wilt disease harbor unique bacterial communities. Plant Soil 393, 21–33. doi: 10.1007/S11104-015-2474-9

[B78] Siegel-Hertz K. Edel-Hermann V. Chapelle E. Terrat S. Raaijmakers J. M. Steinberg C. (2018). Comparative microbiome analysis of a fusarium wilt suppressive soil and a fusarium wilt conducive soil from the châteaurenard region. Front. Microbiol 0. doi: 10.3389/FMICB.2018.00568 PMC5893819 29670584

[B79] Staver C. Pemsl D. E. Scheerer L. Perez Vicente L. Dita M. (2020). Ex ante assessment of returns on research investments to address the impact of fusarium wilt tropical race 4 on global banana production. Front. Plant Sci. 11. doi: 10.3389/FPLS.2020.00844 PMC7357546 32733497

[B80] Thomas P. Sekhar A. C. (2017). Cultivation versus molecular analysis of banana (Musa sp.) shoot-tip tissue reveals enormous diversity of normally uncultivable endophytic bacteria. Microb Ecol. 73, 885–899. doi: 10.1007/S00248-016-0877-7 27833995

[B81] Tripathi L. Ntui V. O. Tripathi J. N. (2019). Application of genetic modification and genome editing for developing climate-smart banana. Food Energy Secur 8, e00168. doi: 10.1002/FES3.168

[B82] Trivedi P. Leach J. E. Tringe S. G. Sa T. Singh B. K. (2020). Plant–microbiome interactions: from community assembly to plant health. Nat. Rev. Microbiol. 18, 607–621. doi: 10.1038/s41579-020-0412-1 32788714

[B83] Vancov T. Keen B. (2009). Amplification of soil fungal community DNA using the ITS86F and ITS4 primers. FEMS Microbiol Lett. 296, 91–96. doi: 10.1111/J.1574-6968.2009.01621.X 19459948

[B84] Viljoen A. Ma L.-J. Molina A. B. (2020). “CHAPTER 8: fusarium wilt (Panama disease) and monoculture in banana production: resurgence of a century-old disease,” in Emerging plant diseases and global food security. The American Phytopathological Society. 159–184. doi: 10.1094/9780890546383.008

[B85] Wang X. Chi Y. Song S. (2024). Important soil microbiota’s effects on plants and soils: a comprehensive 30-year systematic literature review. Front. Microbiol 15. doi: 10.3389/FMICB.2024.1347745/XML/NLM PMC10999704 38591030

[B86] Weber O. B. Muniz C. R. Vitor A. O. Freire F. C. O. Oliveira V. M. (2007). Interaction of endophytic diazotrophic bacteria and Fusarium oxysporum f. sp. cubense on plantlets of banana ‘Maça.’. Plant Soil 298, 47–56. doi: 10.1007/S11104-007-9335-0

[B87] Were E. Viljoen A. Rasche F. (2023). Back to the roots: Understanding banana below-ground interactions is crucial for effective management of Fusarium wilt. Plant Pathol. 72, 19–38. doi: 10.1111/PPA.13641

[B88] Wong C. K. F. Zulperi D. Saidi N. B. Vadamalai G. (2021). A Consortium of Pseudomonas aeruginosa and Trichoderma harzianum for Improving Growth and Induced Biochemical Changes in Fusarium Wilt Infected Bananas. Trop. Life Sci. Res. 32, 23. doi: 10.21315/TLSR2021.32.1.2 33936549 PMC8054672

[B89] Xue C. Penton C. R. Shen Z. Zhang R. Huang Q. Li R.. (2015). Manipulating the banana rhizosphere microbiome for biological control of Panama disease. Sci. Rep. 5, 1–11. doi: 10.1038/SREP11124;TECHMETA=23,49;SUBJMETA=2142,2254,2565,326,514,61,631;KWRD=METAGENOMICS,NEXT-GENERATION+SEQUENCING PMC4525139 26242751

[B90] Ye L. Wang X. Wei S. Zhu Q. He S. Zhou L. (2022). Dynamic analysis of the microbial communities and metabolome of healthy banana rhizosphere soil during one growth cycle. PeerJ 10. doi: 10.7717/PEERJ.14404 PMC9677880 36420134

[B91] Zhou D. Jing T. Chen Y. Wang F. Qi D. Feng R.. (2019). Deciphering microbial diversity associated with Fusarium wilt-diseased and disease-free banana rhizosphere soil. BMC Microbiol 19, 1–13. doi: 10.1186/S12866-019-1531-6/FIGURES/7 31299891 PMC6626388

[B92] Zorrilla-Fontanesi Y. Pauwels L. Panis B. (2020). Strategies to revise agrosystems and breeding to control Fusarium wilt of banana. Nat. Food 1, 599–604. doi: 10.1038/s43016-020-00155-y 37128105

