# Supplementary material for: Identifying bacterial and fungal communities associated with Fusarium-wilt symptomatic and non-symptomatic ‘Gros Michel’ banana plants in Ecuador
Source: Front Cell Infect Microbiol. 2025 Jun 23;15:1572860. doi: 10.3389/fcimb.2025.1572860 (PMC12230051; doi:10.3389/fcimb.2025.1572860)
Supplement: Supplementary file 1 [file DataSheet1.docx]

Supplementary Material

# Supplementary Data

Supplementary Material of the article “Identifying bacterial and fungal communities associated with Fusarium-wilt Symptomatic and Non-symptomatic ‘Gros Michel’ Banana plants in Ecuador”.

## Supplementary Figures


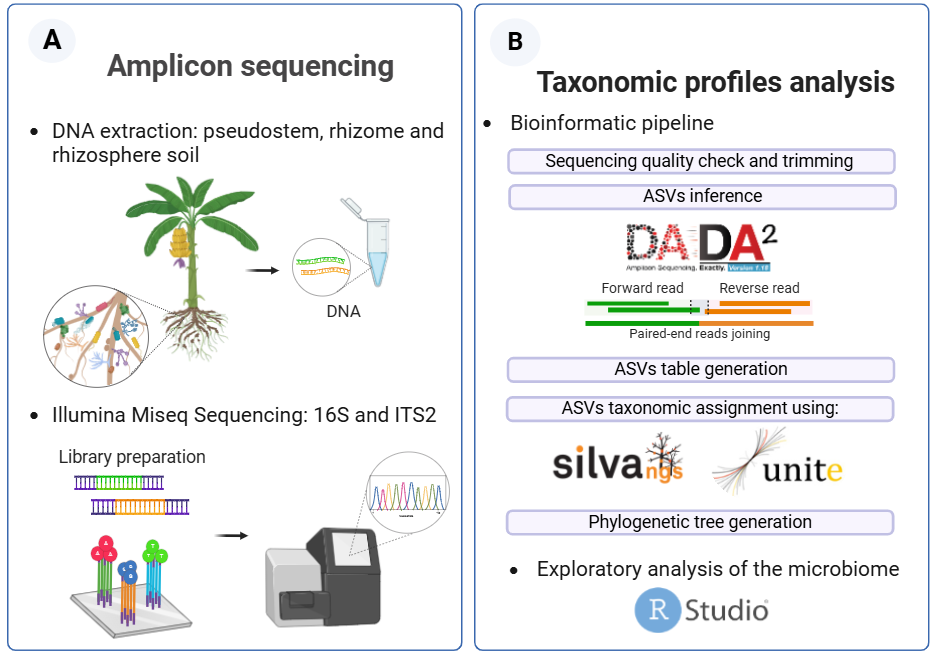


**Supplementary Figure 1.** Workflow of the present investigation. Created in BioRender.com.


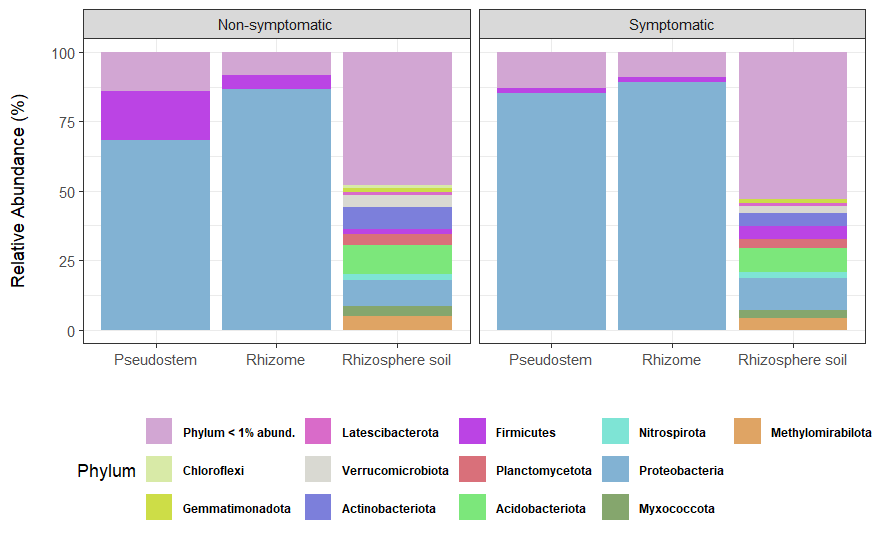


**Supplementary Figure 2.** Relative abundance of bacterial phyla grouped by environment. Taxa with a relative abundance below 1% are not shown.


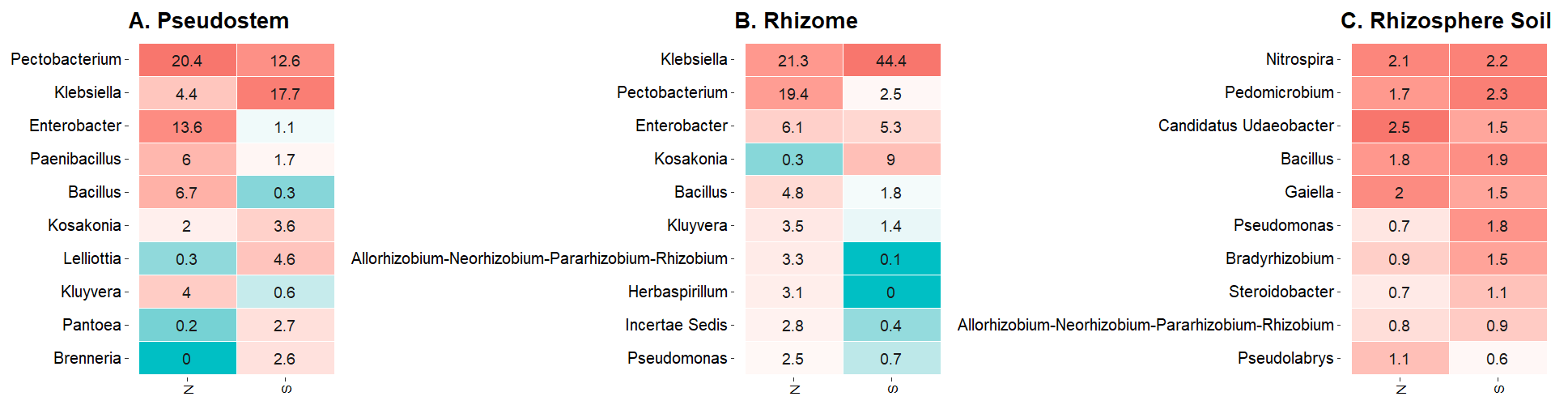


**Supplementary Figure 3.** Heat map of bacterial genera grouped by environment. N= Non-symptomatic, S=Symptomatic.


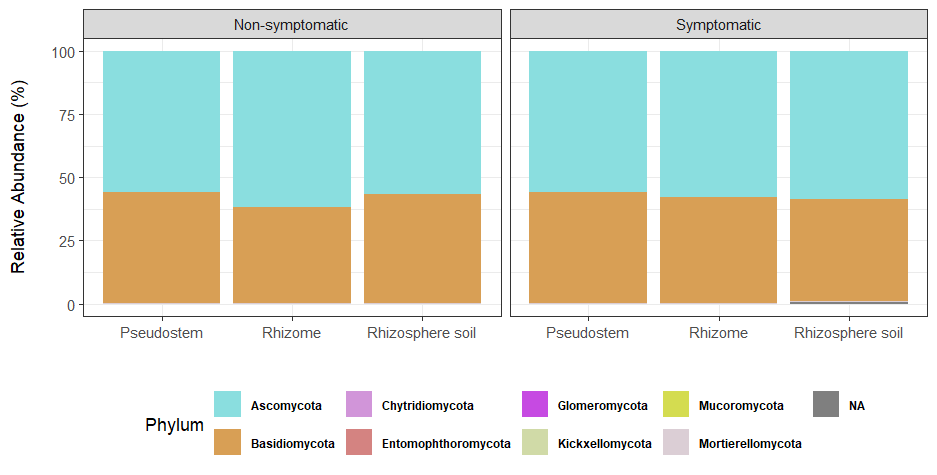


**Supplementary Figure 4.** Relative abundance of fungal phyla grouped by environment. "Not available" (NA) indicates ASVs that could not be taxonomically assigned at the Phylum level.


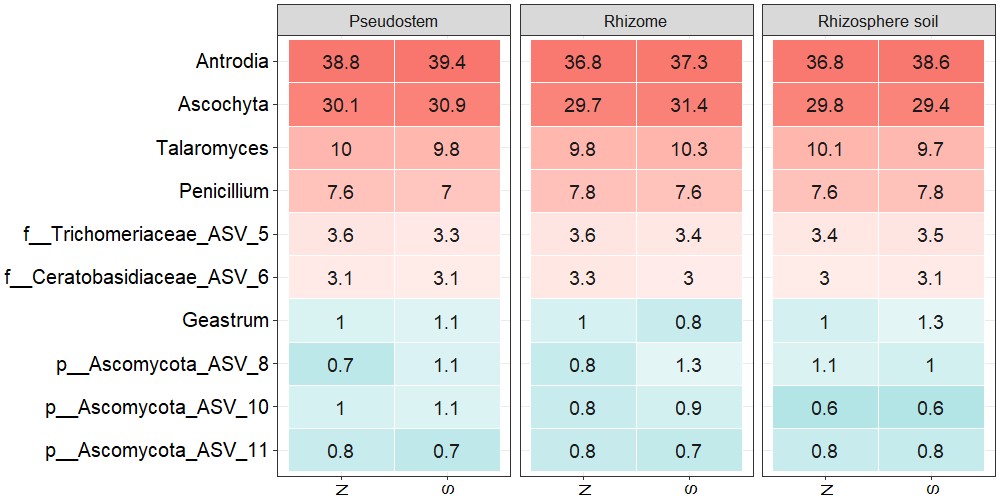


**Supplementary Figure 5.** Heat map of fungal genera grouped by environment. N= Non-symptomatic, S=Symptomatic.


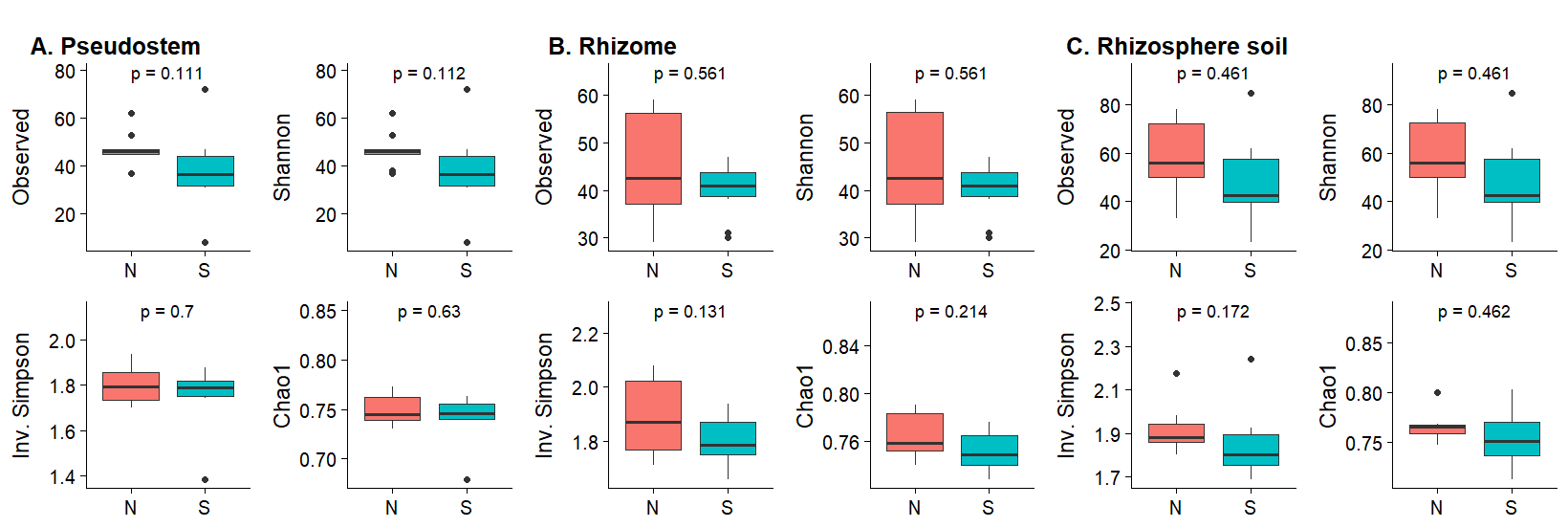


**Supplementary Figure 6.** Box plots of alpha diversity measures for fungal communities based on symptomatology. A. Pseudostem; B. Rhizome; C. Rhizosphere soil. Note: The p-values are indicated at the top of each plot.

# Supplementary Tables

**Supplementary Table 1.** Summary of the amount of data after each read-processing stage

| Stage | Bacteria (16S V3-V4) | | Fungi (ITS2) | |
| --- | --- | --- | --- | --- |
|  | Reads | % Remaining | Reads | % Remaining |
| Input reads | 5411075 | 100 | 4568255 | 100 |
| DADA2: Filtering and trimming | 2973842 | 54.95 | 4566186 | 99.95 |
| DADA2: Dereplication (forward) | 2838889 | 52.46 | 4530144 | 99.17 |
| DADA2: Dereplication (reverse) | 2865520 | 52,96 | 4554525 | 99.70 |
| DADA2: Merging | 2543868 | 47,01 | 4082580 | 89.37 |
| DADA2: Chimera removal | 1750392 | 32,35 | 3824572 | 83.37 |
| Unwanted taxa and uninformative sample removal | 1048265 | 19,37 | 3823325 | 83.69 |
